# Dual Transcriptome and Metabolic Analysis of *Vitis vinifera* cv. Pinot Noir Berry and *Botrytis cinerea* During Quiescence and Egressed Infection

**DOI:** 10.3389/fpls.2019.01704

**Published:** 2020-01-30

**Authors:** Zeraye Mehari Haile, Giulia Malacarne, Stefania Pilati, Paolo Sonego, Marco Moretto, Domenico Masuero, Urska Vrhovsek, Kristof Engelen, Elena Baraldi, Claudio Moser

**Affiliations:** ^1^ Department of Genomics and Biology of Fruit Crops, Research and Innovation Centre, Fondazione Edmund Mach (FEM), San Michele all'Adige, Italy; ^2^ Laboratory of Biotechnology and Plant Pathology, DISTAL, University of Bologna, Bologna, Italy; ^3^ Plant Protection Research Division of Melkassa Agricultural Research Center, Ethiopian Institute of Agricultural Research (EIAR), Addis Ababa, Ethiopia; ^4^ Unit of Computational Biology, Research and Innovation Centre, Fondazione Edmund Mach (FEM), San Michele all'Adige, Italy; ^5^ Department of Food Quality and Nutrition, Research and Innovation Centre, Fondazione Edmund Mach, San Michele all'Adige, Italy; ^6^ ESAT-ELECTA, Electrical Energy and Computer Architectures, Leuven, Belgium

**Keywords:** *Botrytis cinerea*, hard-green berry, ripening, egression, quiescence, *Vitis vinifera*

## Abstract

*Botrytis cinerea* is an important necrotroph in vineyards. Primary infections are mostly initiated by airborne conidia from overwintered sources around bloom, then the fungus remains quiescent from bloom till maturity and egresses at ripeness. We previously described in detail the process of flower infection and quiescence initiation. Here, we complete the characterization studying the cross-talk between the plant and the fungus during pathogen quiescence and egression by an integrated transcriptomic and metabolic analysis of the host and the pathogen. Flowers from fruiting cuttings of the cv. Pinot Noir were inoculated with a GFP-labeled strain of *B. cinerea* at full cap-off stage, and molecular analyses were carried out at 4 weeks post inoculation (wpi, fungal quiescent state) and at 12 wpi (fungal pre-egression and egression states). The expressed fungal transcriptome highlighted that the fungus remodels its cell wall to evade plant chitinases besides undergoing basal metabolic activities. Berries responded by differentially regulating genes encoding for different PR proteins and genes involved in monolignol, flavonoid, and stilbenoid biosynthesis pathways. At 12 wpi, the transcriptome of *B. cinerea* in the pre-egressed samples showed that virulence-related genes were expressed, suggesting infection process was initiated. The egressed *B. cinerea* expressed almost all virulence and growth related genes that enabled the pathogen to colonize the berries. In response to egression, ripe berries reprogrammed different defense responses, though futile. Examples are activation of membrane localized kinases, stilbene synthases, and other PR proteins related to SA and JA-mediated responses. Our results indicated that hard-green berries defense program was capable to hamper *B. cinerea* growth. However, ripening associated fruit cell wall self-disassembly together with high humidity created the opportunity for the fungus to egress and cause bunch rot.

## Introduction

*Botrytis cinerea* is a necrotrophic fungus responsible for significant economic losses in vineyards by causing bunch rot. The disease is mostly observed on ripe berries, following rainfalls or a long period of high humidity close to harvest, and develops into gray mold. Primary infections are usually initiated by airborne conidia from overwintered sources (Nair et al., 1995; Elmer and Michailides, 2004) and mostly occur at bloom leading to quiescent infection (McClellan and Hewitt, 1973; Keller et al., 2003; Pezet et al., 2003b). Quiescent infection is an interesting phenomenon in *B. cinerea–*plant interaction where the pathogen spends prolonged time in the host tissue asymptomatically, without being aggressive (Williamson et al., 1987; McNicol and Williamson, 1989; Coertze and Holz, 2002; Shaw et al., 2016). Recently, it has been observed in grapevine that *B. cinerea* inoculated at full bloom stays quiescent until full maturity and egresses at ripening causing bunch rot (Haile et al., 2017; Haile et al., 2019).

What drives and keeps *B. cinerea* into quiescence until berry ripening is not fully known, but preformed and induced defense mechanisms, including immature berries skin features such as polyphenols in the berry skin cell wall and the thickness of the epidermal cell layer complex, have been proposed as part of the ontogenic resistance to *B. cinerea* (Goetz et al., 1999; Keller et al., 2003; Deytieux-Belleau et al., 2009). Molecular analysis has shown that upon contact with the grapevine flower, *B. cinerea* induces genes, both encoding known virulence factors, such as boctinic acid (*BcBOA6*), botrydial phytotoxin (*BcBOT1 and BcBOT2*), polygalacturonase 2 (*BcPG2*), and superoxide dismutase 1 (*BcSOD1*) encoding genes, and contributing to the infection program, such as oxaloacetate acetyl hydrolase (*BcOAH*), endo-β-1,4-xylanase (*BcXYN11A*), and glutathione S-transferase (*BcGST1*) encoding genes, to cause disease (Haile et al., 2017). However, no visible disease progress was observed despite the confirmed presence of the pathogen on the immature berries (Haile et al., 2017). As a response to the infection attempt, grapevine flowers react by reprogramming the expression of genes encoding antimicrobial proteins, monolignol biosynthesis (*VvPAL*, *VvCOMT*, *VvCCoAMT*, and *VvCAD*), stilbenoids (*VvSTSs*), and prompting oxidative burst (*VvGLP3*). These induced defense responses of the grapevine flowers are presumably involved in *B. cinerea* quiescence (Haile et al., 2017). Another study highlighted the involvement of the salicylic acid (SA) dependent defense pathway together with the accumulation of ROS and the activation of stilbene and lignin biosynthesis as main factors arresting *B. cinerea* progress on véraison berries but not in the ripe ones, that were fully susceptible to the pathogen (Kelloniemi et al., 2015).

The transition from a quiescent to an active infection mostly occurs during fruit ripening. Physiological and biochemical changes that occur in the fruit during ripening, together with favorable climatic conditions during ripening, are suggested to trigger the transition (Prusky, 1996; Barnes and Shaw, 2002; Prusky et al., 2013). Cell wall loosening and appearance of disassembled cell wall substrates (Cantu et al., 2008), decrease in preformed and inducible host defense responses and change in hormonal balance and pH (Prusky, 1996; Prusky et al., 2013) are the major events during berry ripening that could enhance the egression and outgrowth of a quiescent necrotrophic pathogen. Egression impairs product quantity, quality, and appearance.

Global expression profiling of both pathogen and host at quiescent and egression stages of the infection enables to gain insights into signaling, metabolic pathways, transcriptional control, and defense responses involved in the cross-talk. Here we report the simultaneous transcriptome and secondary metabolite analyses of the *B. cinerea–*grapevine pathosystem, at hard-green and ripe stages, after host inoculation with *B. cinerea* conidia at full cap-off stage. Our results revealed that grapevine berries were able to keep the fungus quiescent for 12 weeks upon flower inoculation activating defense responses similar to the ones activated at bloom (Haile et al., 2017). On the other side, the pathogen was able to maintain its basal metabolic activities during quiescence and cause disease when the fruit activates some physiological responses which favor its egression, i.e. at ripening. These new molecular evidences represent a valuable resource in order to define the most appropriate infection stages for treatments against *B. cinerea*. 

## Materials and Methods

### Fungal Isolate, Plant Material, and Inoculation

A genetically transformed *B. cinerea* strain, B05.10, expressing a green fluorescent protein (GFP) was used as in Haile et al., 2017. Grapevine fruiting cuttings obtained from Pinot Noir winter woody cuttings were grown and infected as described in Haile et al., 2017.

Flowers at full cap*–*fall stage [EL25/26, according to Eichorn and Lorenz (1977)] were inoculated by placing a 1.5 µl droplet of either conidia solution of GFP-labeled B05.10 (2*10^5^ ml^–1^) or distilled water (mock inoculation) close to the receptacle area. Conidia were obtained from *B. cinerea* grown on PDA at 25°C for 10 days, and the concentration was determined using a hemacytometer under light microscope. Inoculation was made on three biological replicates, considering the inflorescence from a fruiting cutting as one biological replicate. After inoculation, the whole pot was immediately bagged in a clear plastic bag sprayed with water, for 24 h, in order to ensure high humidity around the inoculated inflorescence, which is an essential factor for conidial germination. Inoculated inflorescences were regularly inspected for gray mold growth until fruit ripening. At full coloring (approximately 10 weeks post inoculation, wpi), bunches were bagged for 2 weeks with plastic bags, to create favorable humidity for *B. cinerea* to egress.

Samples were collected at two time points, at 4 wpi (hard green berries), and at 12 wpi (ripe berries) when *Botrytis* egression was evident on a subset of berries. For the latter time point, two kinds of samples were collected: berries with visible egressed *Botrytis* and berries without visible *Botrytis* sign from a cluster. Samples without visible *Botrytis* signs are hereafter called berries with “pre-egressed” *Botrytis*, while the others are called berries with “egressed” *Botrytis.* Samples were snap frozen in liquid nitrogen and stored at -80 °C until use. For transcriptome and polyphenol analyses, fleshy exocarp was excised from individual fruits at the site of inoculation or where *Botrytis* symptoms were visible. 

### RNA Extraction, RNA-Seq, and qPCR Analyses

Extraction of RNA was performed as described in Haile et al., 2017. Single-end reads of 100 bp long sequences were obtained for each sample using a Next Generation Sequencing Platform HiSeq 1500 (Illumina, San Diego, CA). Approximately 20 million strand-specific sequences were obtained, except for pre-egressed samples (above 45 million), where the sequence depth was doubled in order to obtain more reads of *Botrytis* origin. The quality of the reads was checked using FastQC (version 0.11.2) software and pre-processed by cutadapt [version 1.8.1; Martin (2011)] for adapter. Genome assemblies of grapevine (12Xv1, http://genomes.cribi.unipd.it/) and *B. cinerea* (strain B05.10) (ASM83294v1, http://fungi.ensembl.org) were used as reference sequences. The alignment was made by Subread aligner (Liao et al., 2013) and raw read counts were extracted using the featureCount read summarization program (Liao et al., 2014). All raw RNA-Seq read data are deposited in the NCBI Short Read Archive (http://www.ncbi.nlm.nih.gov/sra/) under the BioProject accession code PRJNA414966.

The RNA sequences of *B. cinerea* (B05.10), from the PDB cultured conidia (used in Haile et al., 2017), were also used in this study as a control for determining the *in planta Botrytis* transcripts.

Finally, cDNA synthesis and quantitative polymerase chain reaction (qPCR) assay were carried out as described in Haile et al., 2017. For qPCR assay, each amplification reaction was run in triplicate, and *VvACT* and *VvGAPDH*, and *BcRPL5* and *BcTUBA* genes were selected using GeNORM (Vandesompele et al., 2002) as reference genes for grapevine and *B. cinerea* gene expression normalization, respectively. Amplification efficiencies of each primer pair were calculated with LinReg software (Ruijter et al., 2009). The obtained amplification efficiency was used to calculate the relative quantity (RQ) and normalized relative quantity (NRQ) according to Hellemans et al. (2007). All primers and corresponding gene identifiers are listed in **Supplemental Table S1**. 

### Polyphenol Extraction and Analysis

Extraction of polyphenol and ultra high performance liquid chromatography–diode array detection–mass spectrometry (UHPLC–DAD–MSMS) analysis were carried out as described in Haile et al., 2017. 

### Statistical Analysis

#### qPCR Data

Statistical analyses of the qPCR results were made after log2(NRQ) transformation (Rieu and Powers, 2009). Statistical significance was calculated by Tukey’s honestly significant difference test or an unpaired heteroscedastic Student’s *t* test, considering each technical replicate as an individual sample. 

#### RNA-Seq Data

For differential expression analysis, the voom method (Law et al., 2014) was applied to estimate the mean–variance relationship of the log-counts and produce a precision weight for each observation that was fed into the limma empirical Bayes analysis pipeline.(Smyth, 2004). Two-sample *t*-test was used for transcripts of grapevine at 4 wpi (mock inoculated vs *Botrytis* inoculated) and *B. cinerea* at egression (PDB-cultured *Botrytis* vs egressed *Botrytis*), whereas one-way ANOVA for grapevine transcripts at 12 wpi (mock inoculated vs pre-egressed *Botrytis* vs egressed *Botrytis*). Genes were considered differentially expressed (DE) if they fulfill a *p*-value of < 0.01 and an absolute fold change of ≥ 2.0.

Principal component analysis (PCA) was performed using prcomp function in R on scale-normalized counts. *K*-means clustering of differentially expressed genes based on fold change values (using cosine distance) was performed using the kmeans function in R. 

### Functional Classification Based on Gene Ontology, Vitisnet, and Mapman

Grapevine DE genes were subjected to enrichment analyses using: (1) VitisNet annotation within the VESPUCCI grapevine gene expression compendium (http://vespucci.colombos.fmach.it) (Grimplet et al., 2012; Moretto et al., 2016), *p*-value < 0.01; (2) customized GO annotation and annotated reference, taken from CRIBI annotation (http://www.cribi.unipd.it/), using AgriGO analysis tool (http://bioinfo.cau.edu.cn/agriGO/analysis.php; Du et al., 2010). Enriched GO terms (FDR <0.01) were visualized using the ‘Reduce + Visualize Gene Ontology’ (REViGO) webserver (http://revigo.irb.hr; Supek et al., 2011). Additionally, the differentially expressed genes were visualized in the context of biotic stress pathway using the GrapeGen 12Xv1 annotations version (Lijavetzky et al., 2012) with the help of MapMan tool (Thimm et al., 2004).

## Results

### *Botrytis cinerea* Inoculation of Grapevine

**Figure 1:**
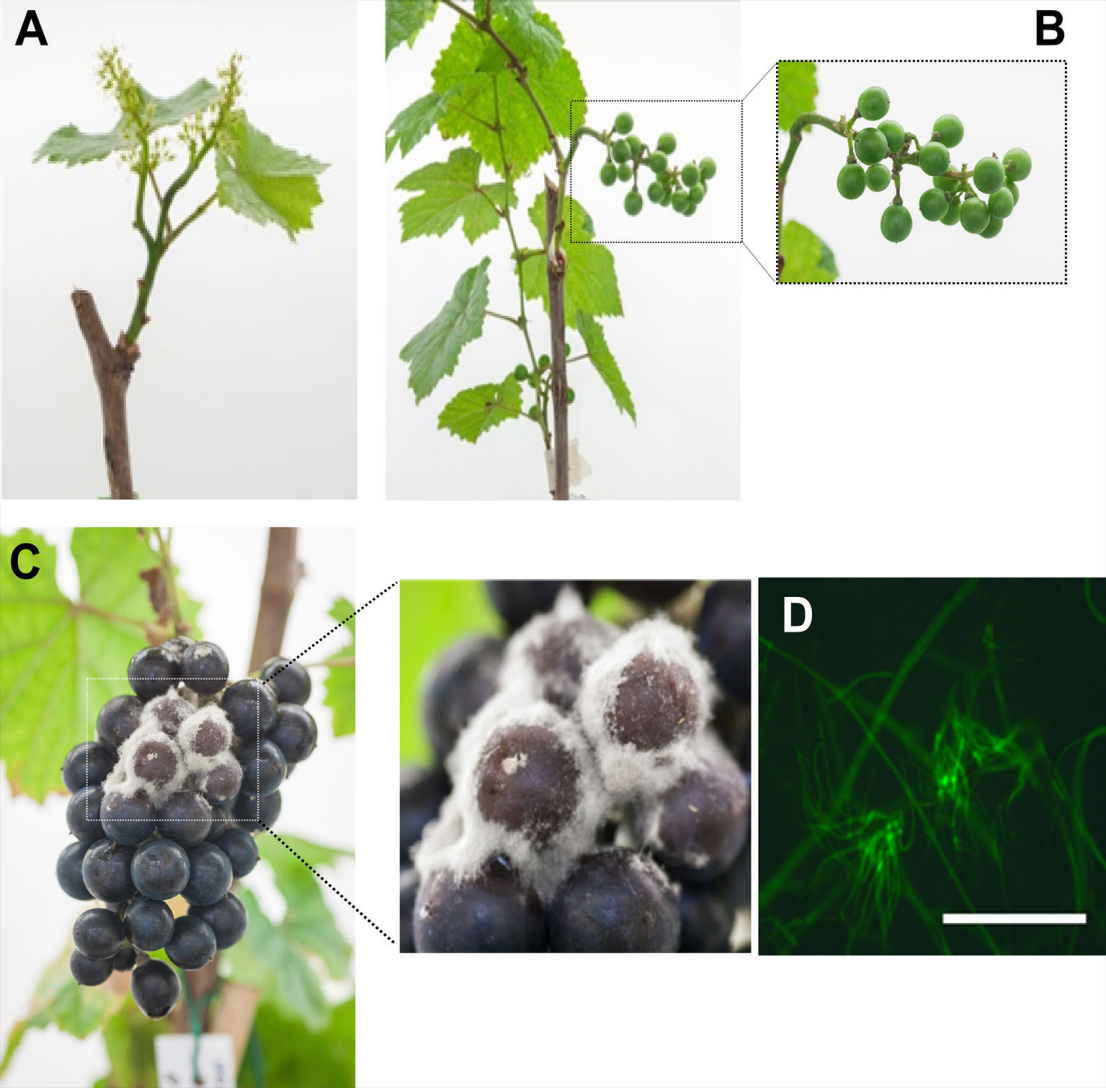
*Botrytis cinerea* infected grapevine flowers and their development until berry ripening. **(A)** Flowers 24 hours post inoculation with GFP-labelled B05.10 strain at full cap-fall stage (EL25/26). **(B)** Healthy looking, asymptomatic, hard-green berries at 4 weeks post inoculation (wpi). **(C)** Egression of *B. cinerea* at ripening (12 wpi). **(D)** Fluorescence of mass of mycelia isolated from the outgrown *B. cinerea*; *white bar* represents 50 µm.

**Figure 2:**
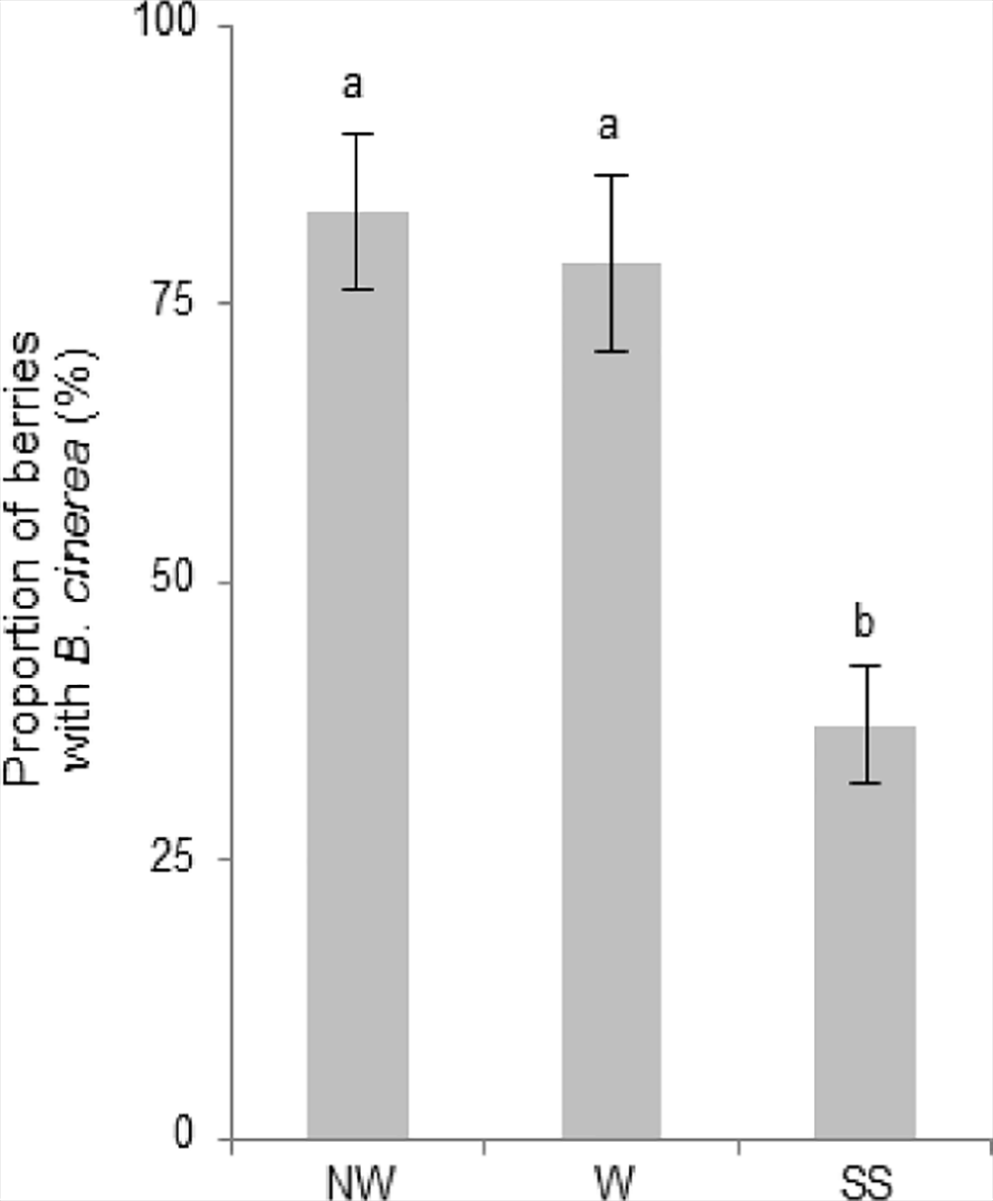
Proportion of berries showing *B. cinerea* at 4 wpi, following flower inoculation with the fungus, as determined by plating out. Plating out was made on selective media (PDA with Hygromycin B, 70 µg/ml) to check the presence of quiescent *B. cinerea* before (*NW*) or after washing (*W*), or after surface sterilization (*SS*). Mean proportion of berries (8–10 berries from each of six biological replicates) showing GFP-labeled B05.10 growth on the selective media. Error bars indicate standard error. Mean proportions labeled with the same letter are significantly not different, according to Tukey’s honestly significant difference test (*P* ≤ 0.05), using one-way ANOVA.

Grapevine flowers were inoculated with a GFP-labeled B05.10 strain at full cap-off stage by placing 300 conidia around the receptacle area, and the infection was monitored until ripening, for 12 weeks (**Figure 1**). No visible symptoms or sign of the fungus growth were observed until full coloring. The proportion of berries carrying *B. cinerea* at hard-green berry stage (4 wpi; **Figure 1B**) derived from flower inoculation was checked by plating out on selective media (**Figure 2**). *B. cinerea* was present quiescently on 80% of the asymptomatic berries in the samples washed or not with water; however, the proportion dropped to 40% when the berries were surface sterilized, suggesting that the fungus mostly resides in the first few outer epidermal cell layers, those affected by the sterilization procedure. At 10 wpi, at full color change, bunches were bagged with plastic bags to increase humidity and favor *B. cinerea* growth (Barnes and Shaw, 2002; Elmer and Michailides, 2004; Carisse, 2016). Two weeks later, egression of *B. cinerea* was observed (**Figure 1C**), and cross checking the strain using fluorescence microscopy confirmed that the strain was the GFP-labeled B05.10 inoculated at cap-off stage (**Figure 1D**).

### Dual Transcriptomic Analysis of Grape Berries and *B. cinerea* During Their Interaction

Hard green (4 wpi) and ripe berries (12 wpi), which were mock- and *Botrytis*-inoculated at cap-off stage, were harvested in three biological replicates for dual (plant and fungus) transcriptome analysis using the RNA-seq method. The fraction of reads uniquely mapped to the *Vitis vinifera* reference genome ranged from 13 to 88%, the smaller proportion being from samples with egressed *B. cinerea*. The fraction of reads uniquely mapped to *B. cinerea* reference genome was below 1% for the 4 wpi and pre-egressed samples, up to 67% for the *B. cinerea* egressed samples, and up to 78% for PDB grown *Botrytis* samples (**Supplemental Table S2**).

**Figure 3:**
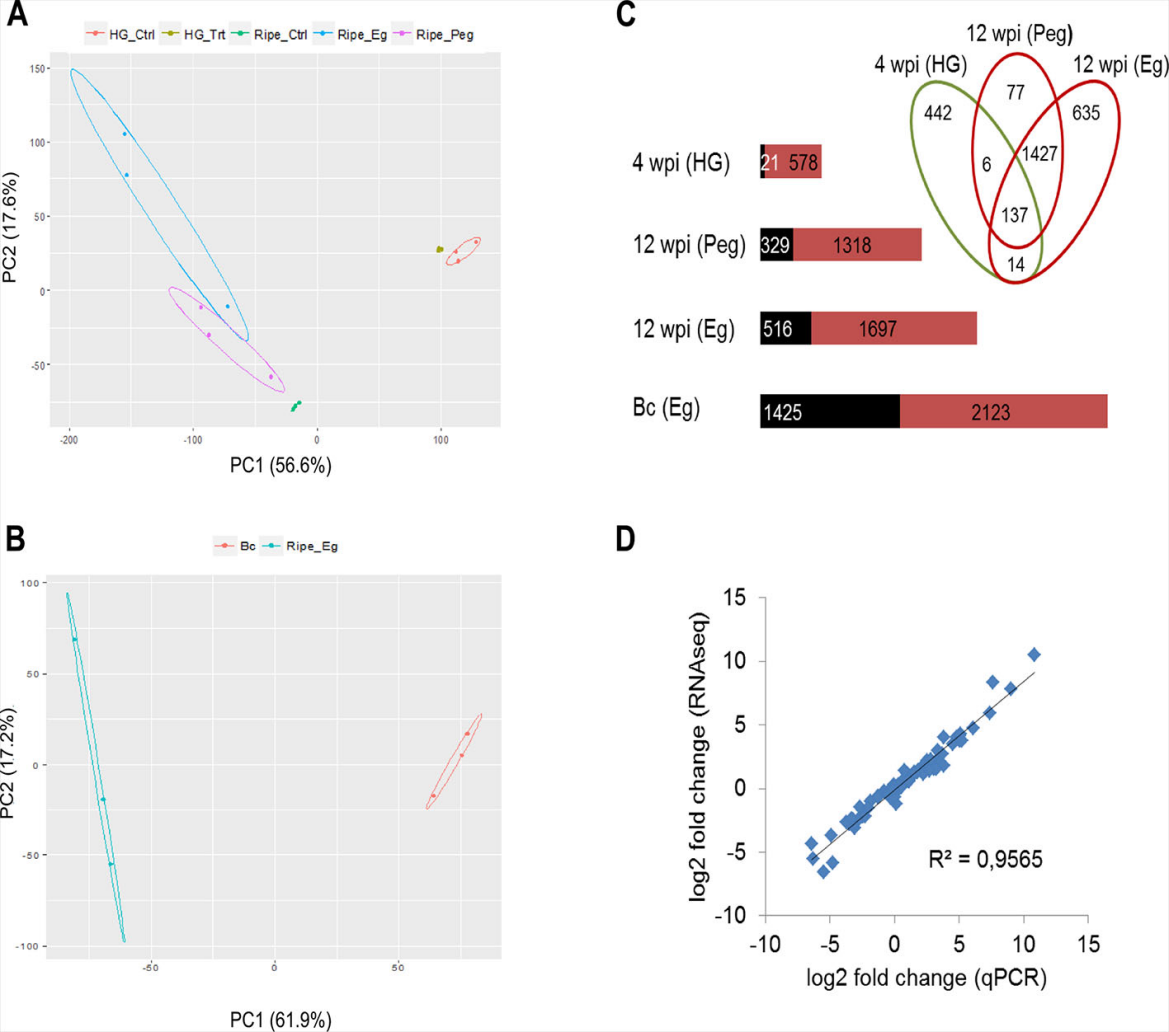
Global evaluation of the RNA-seq data and of the differentially expressed (DE) genes. PCA displaying the biological variations among samples, for grapevine genes **(A)** and *B. cinerea* genes **(B)**. *Ctrl*, mock inoculated; *Trt*, *B. cinerea* inoculated; *Bc*, *Botrytis cinerea*; *HG*, hard-green berry; *Eg*, *B. cinerea* egression state; *Peg*, *B. cinerea* pre-egression state. Raw count data were used after precision weight was calculated by the voom method (Law et al., 2014). **(C)** Number of DE genes (*P* < 0.01, absolute fold change > 2.0) upon *B. cinerea* infection at 4 weeks post inoculation (wpi) and 12 wpi in grape berry and during egression in *B. cinerea*; upregulated genes (*red*) and downregulated genes (*black*). The Venn diagram was generated using Venny v2.1.0 (Oliveros, 2015). *Bc*, *Botrytis cinerea*; *Eg*, egression; *HG*, hard green; *Peg*, pre-egession. **(D)** Correlation of gene expression values obtained by RNA-seq and qPCR. Relative expression levels were calculated for 18 *Vitis* genes and an R^2^ value of 0.96 was obtained comparing the results obtained with the two techniques.

The biological variability of all the samples was assessed using PCA on the gene expression data. Concerning grapevine data, samples were largely separated by growth stage along the first principal component, but within each growth stage most of the variation in gene expression was explained by the infection process (**Figure 3A**). With regard to *B. cinerea*, the PCA highlighted remarkable difference in gene expression between the egression stage on berries at ripening and the growth in liquid PDB medium (**Figure 3B**).

For grapevine genes, differential expression was computed between *Botrytis*- and mock-inoculated berries imposing a *p*-value < 0.01 and an absolute fold change > 2 (**Supplemental Figure S1**). At 4 wpi, 599 genes of grapevine were differentially expressed (DE) due to *B. cinerea* infection, whereas the number increased to 2,296 at 12 wpi (**Figure 3C**, **Supplemental Tables S3** and **S4**). Only 157 genes were in common, suggesting that the host response is quite different at molecular level at the two growth stages. Concerning *B. cinerea* genes, for samples at 4 wpi and at 12 wpi (pre-egressed), it was not possible to compute DE genes due to limited amount of fungal RNA in the samples (**Supplemental Tables S6** and **S7**). However, for the egression stage 3,548 DE genes were obtained from the comparison with PDB cultured *Botrytis* (**Figure 3C** and **Supplemental Table S5**). To gain more information from the samples at 4wpi and 12 wpi (pre-egressed), qPCR assay was used to study the expression profile of selected fungal genes.

The gene expression values obtained from RNA-seq were validated using qPCR assay. To this end, the expression of 18 grapevine genes having different expression profile from RNA-seq (**Supplemental Table S8**) were analyzed and a strong correlation (*R* ^2^ = 0.96) was observed between the results obtained with the two techniques (**Figure 3D**).

**Figure 4:**
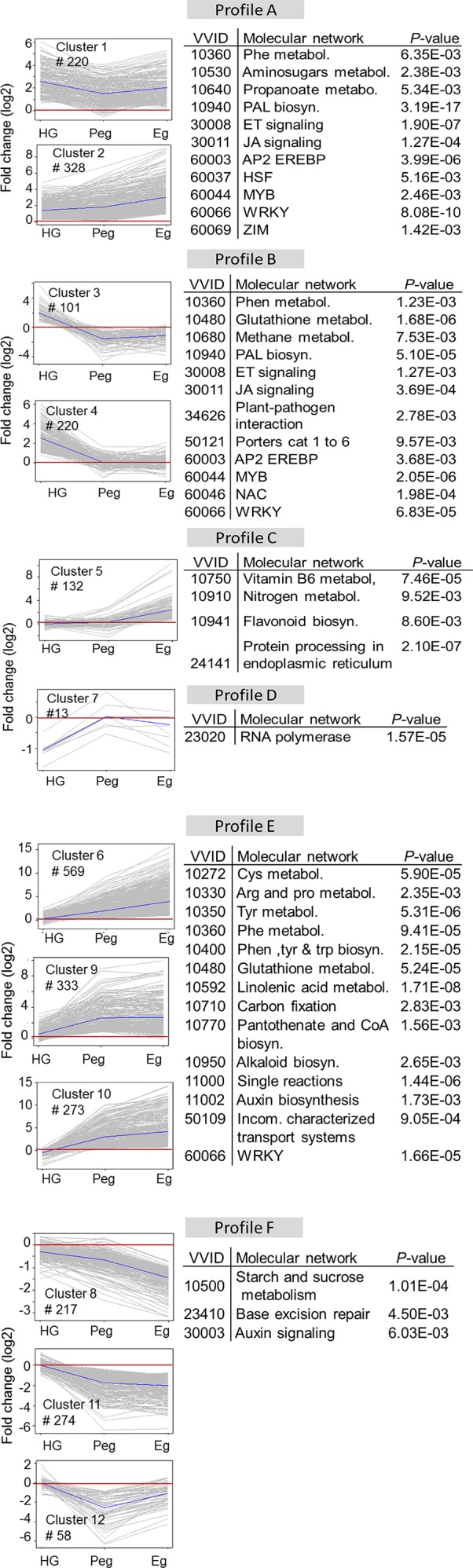
Profiles of grapevine berry trancripts at hard-green and ripe stages in response to *B. cinerea* inoculation. K-means clustering of grapevine genes based on the cosine distance of their log2 (fold change) values. Genes that showed at least twofold expression difference with *P*-value < 0.01 were considered, and clustered into 12 clusters. The clusters were grouped into six major profiles (A, B, C, D, E, and F). Molecular enrichment analysis based on VitisNet is provided for each group.

The total number of DE genes of the grapevine berries (2,738), considering both hard-green and ripe stages, were grouped into 12 distinct clusters according to their expression pattern, which can be grouped in six major expression profiles (**Figure 4**). Profile A comprised cluster 1 and 2, where almost all of the genes were induced both in hard-green and ripe stages, albeit with various extent. Genes in profile B, combining cluster 3 and 4, exhibited an induced expression trend due to quiescent *B. cinerea* in the hard-green berry but down-regulated or unaffected during pre-egression and egression stages of the pathogen at ripening. Profile C (cluster 5) included genes whose expression was not affected during *B. cinerea* quiescence in the hard green berry and pre-egressing at ripe stage, however, they were induced during egression. Genes in profile D (cluster 7, having only 13 genes) were down-regulated at hard-green and not affected at ripe stages. Majority of the genes in profile E were up-regulated during pre-egression and egression at ripening, while the opposite was the case for genes in profile F, where in both profiles the genes were not affected by quiescent *B. cinerea* in hard-green berry.

The molecular network enrichment analysis of the gene set of each profile, based on VitisNet annotation, showed an abundance of transcripts in functional classes which are usually affected by biotic stress (**Figure 4**). A considerable number of genes annotated as belonging to phenylpropanoid biosynthesis, phytohormone signaling, and encoding transcriptional factors (TF) were mainly represented in profile A and B; genes involved in protein processing in profile C; genes of amino acid and glutathione metabolisms in profile E; and genes of carbohydrate metabolism in profile F.

### Transcriptional Alterations of *B. cinerea* During Quiescent Infection, at 4 wpi

**Figure 5:**
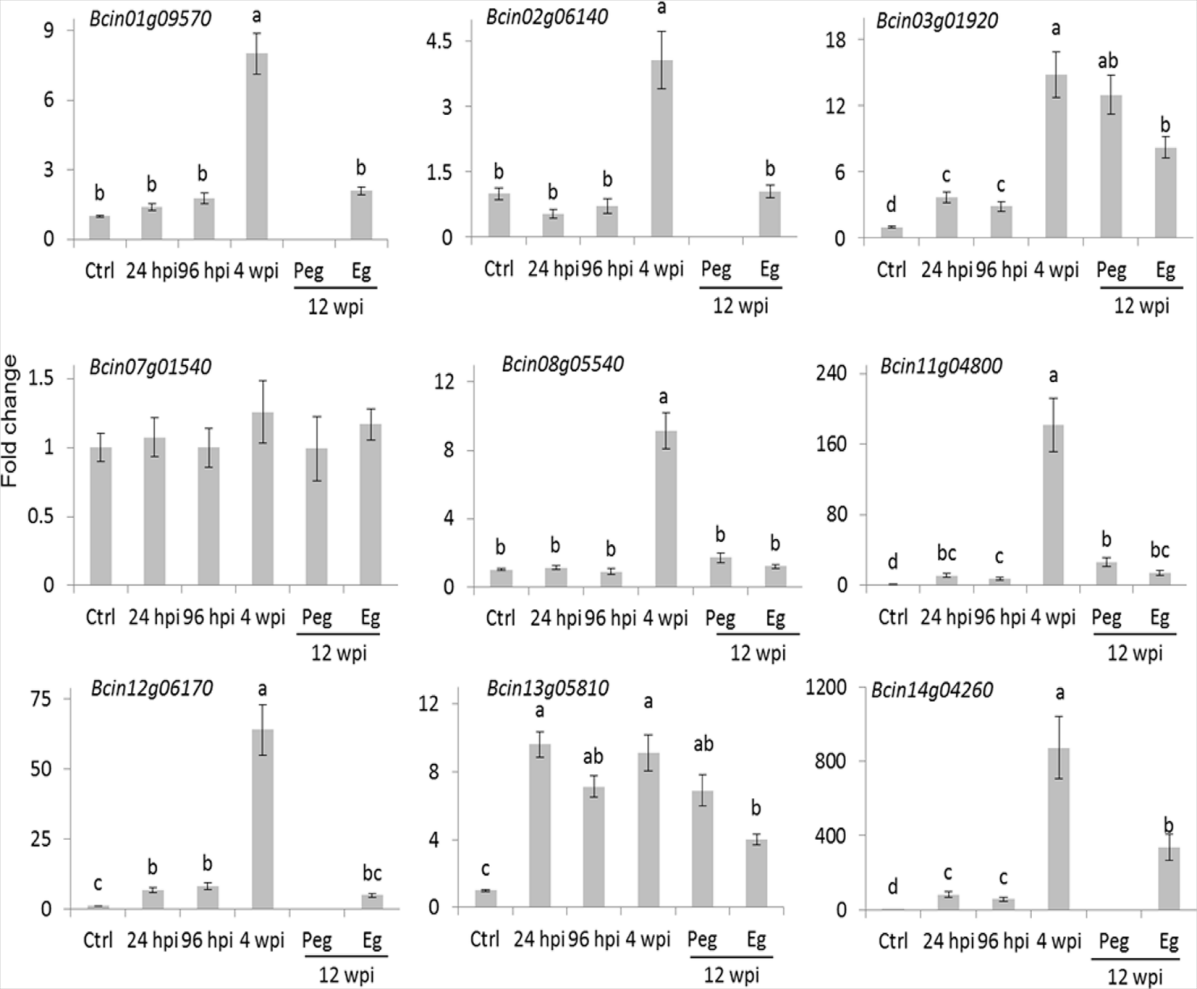
Expression profile of selected *B. cinerea* genes having higher raw reads at hard-green berry stage relative to PDB-cultured *B. cinerea* expression. *Bars* represent fold change of samples at 24 and 96 hours post inoculation (hpi; flower infection), 4 weeks post inoculation (wpi; quiescent infection at hard-green berry stage), and 12 wpi (pre-egression, *Peg*, and egression, *Eg*, stages of *B. cinerea* at ripening) relative to the PDB-cultured *B. cinerea* (*Ctrl*) expression. Normalization based on the expression levels of ribosomal protein L5, BcRPL5, and α tubulin, BcTUBA, was carried out before calculating fold changes. Error bar represents standard error of the mean of three biological replicates. Expression values followed by a *common letter* are significantly not different between samples, according to Tukey’s honestly significant difference test (*P* ≤ 0.05), using one-way ANOVA of log2(NRQ).

The number of reads mapping on the *B. cinerea* transcriptome detected in the inoculated samples at 4 wpi was very low. The reason could largely be linked to a reduced fungal biological activity as well as to the little fungal mass present at the quiescent stage. Only about 20% of the *B. cinerea* genes (1,926) had at least one raw read in all of the three biological replicates. Within this set, those represented by an average of at least ten reads (only 289) were considered as *in planta* expressed fungal transcript and they were functionally annotated using Blast2GO (Conesa et al., 2005) and Amselem et al. (2011) (**Supplemental Table S6**). Using the Combined Graph Function of Blast2GO, the GO slim terms metabolic processes, structural constituent of ribosome, and intracellular were mostly represented in the 289 genes (**Supplemental Table S9**). Fifteen genes from this group, selected on the basis of their function, are presented in **Table 1**. The expression profile of nine of them, involved in functions such as cell wall metabolism, redox-reaction, and transcriptional regulation, was further examined using qPCR assay (**Figure 5**). As depicted in **Figure 5**, all with the exception of *Bcin07g01540* and *Bcin13g05810* had a higher relative expression during quiescent infection at hard green stage than during initial infection, pre-egression, and egression stages.

**Table 1:** Selected *B. cinerea* genes expressed *in planta* in the hard-green berry (4 wpi), with different functions, with their raw RNA-seq reads.

Gene ID	Function (Blast2GO)	RNA-seq reads (average)					PDB culture
		24 hpi	96 hpi	4 wpi	12 wpi		
					Ripe_Peg*	Ripe_ Eg	
Bcin01g09570	yt521-b-like splicing	9	8	15	119	2.69	2.18
Bcin02g06140	CP2 transcription factor protein	2	6	12	2	1.77	3.19
Bcin02g06930	1,3-beta-glucan synthase	49	27	20	24	15.89	12.32
Bcin03g01920	Catalase	19	6	32	12	7.58	1.31
Bcin03g07670	NAD-specific glutamate dehydrogenase	30	20	31	10	2.94	11.14
Bcin07g01540	Elongation factor 2	114	68	42	25	21.91	35.27
Bcin07g03980	Chitin- domain 3	22	12	67	7	3.78	2943
Bcin08g05540	ASG1; CND1, similar to Gas1-like protein	129	54	410	35	24.76	35.78
Bcin09g00200	Glucan endo-1,3-beta-glucosidase	15	15	31	8	5.58	8.51
Bcin11g04800	Chitin deacetylase	10	3	67	4	2.39	210
Bcin11g04930	Stress response ish1	7	6	21	19	15.61	348
Bcin12g06170	Allergen; Peptide transport PTR2	8	2	20	1	682	86
Bcin13g05580	Alcohol dehydrogenase 1	100	47	21	4	4.20	37
Bcin13g05810	Aldehyde dehydrogenase	238	46	48	25	10.69	4.57
Bcin14g04260	Cell surface, Gas2	18	4	112	23	21.02	41

*The sequence depth of the samples was double than the rest.

The values at 4 wpi are highlighted with a gray box.

These results suggested that the biological activities of the fungus were not totally switched off during quiescent infection. The expression of *Bcin01g09570* and *Bcin07g01540* genes, which encode putative yt521-b-like splicing and elongation factor 2 proteins, respectively, and the numerous genes encoding for ribosomal proteins (**Table 1** and **Supplemental Table S6**) indicated instead that protein synthesis activities were carried out during quiescent infection stage. Moreover, the expression of stress and defense related genes such as *Bcin12g06170,* encoding a protein similar to an allergen, and *Bcin11g04800*, encoding a putative chitin deacetylase protein, highlighted that the interaction between the pathogen and the plant was not passive. Chitin deacetylase activity has been speculated to be involved in protecting the fungal cell wall from degradation by plant chitinases (Deising and Siegrist, 1995; El Gueddari et al., 2002). Moreover, *Bcin08g05540*, encoding putative CND1 protein, *Bcin14g04260*, annotated as a putative cell surface protein and Gas2, and *Bcin02g06140,* encoding a putative CP2 transcription factor protein, appeared to be expressed more during quiescent infection at 4 wpi as compared to initial and egression stages of infection (**Figure 5**). These proteins should be involved in maintaining cell wall integrity (Garrett-Engele et al., 1995; Paré et al., 2012).

In addition to cell wall remodeling in which the fungus was engaged, the involvement of genes detoxifying alcohols, aldehydes, and ROS such as *Bcin13g05810, Bcin13g05580,* and *Bcin03g01920* (**Table 1** and **Supplemental Table S6**) during quiescent state indicated that the fungal cell was trying to overcome stresses. In fungal cells, it is known that stress causes ROS production that can lead to aldehydes and alcohols accumulation (Asiimwe et al., 2012).

In summary, the extremely low number of *B. cinerea* genes expressed *in planta* seconded by the absence of any macroscopically noticeable disease progress at 4 wpi suggests that the pathogen was at its basal metabolic activity, but some specific stress related functions were in place.

### Response of Hard-Green Berries to Quiescent *B. cinerea*, at 4 wpi

**Figure 6:**
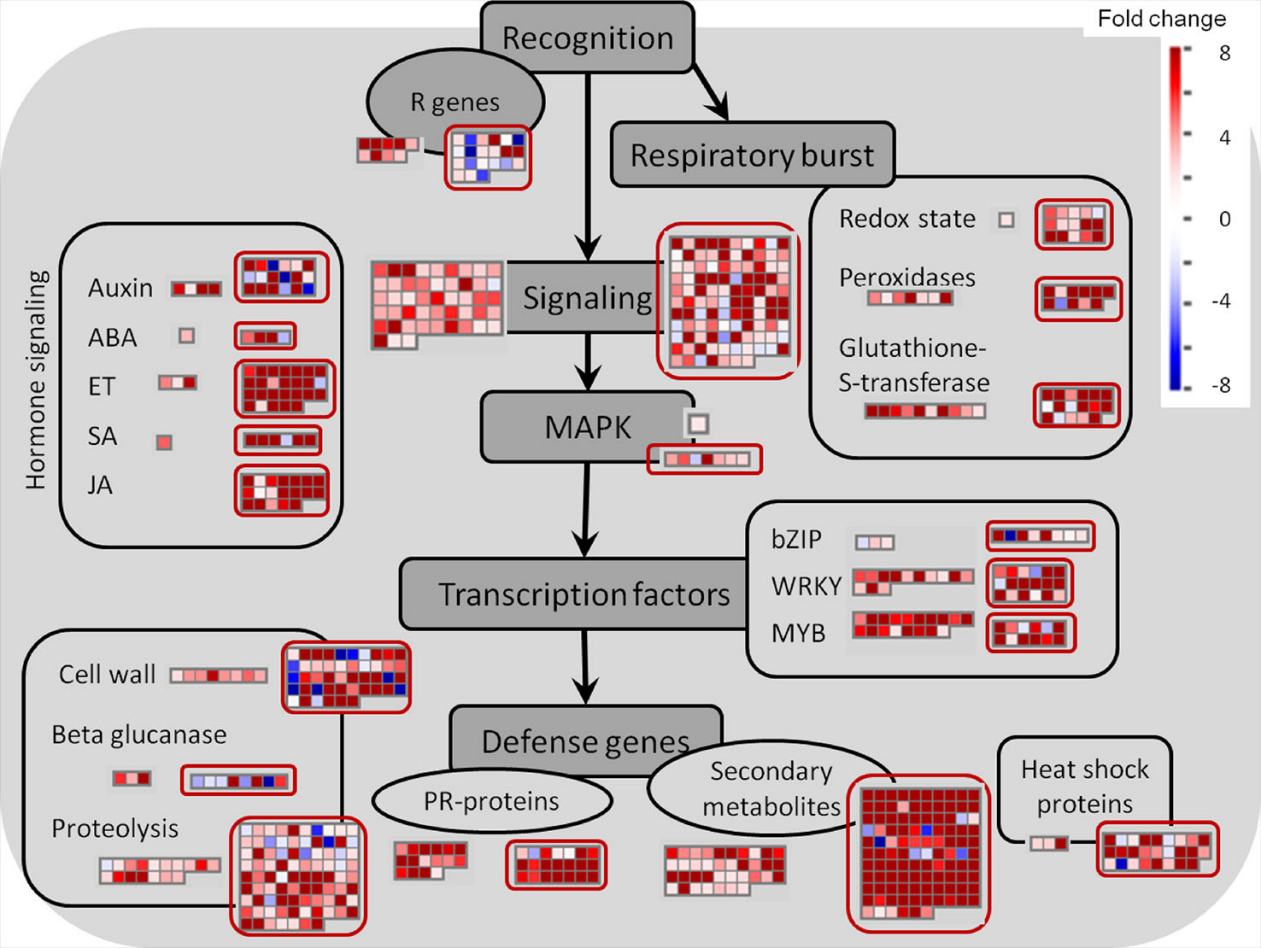
Biotic stress overview of hard-green and ripe berries (*red circles*) due to quiescent and egressed *B. cinerea* infection, respectively, as visualized by MapMan. Up-regulated and down-regulated genes are shown in *red* and *blue*, respectively. The scale bar displays fold change values. ABA, abscisic acid; ET, ethylene; JA, jasmonic acid; MAPK, mitogen-activated protein kinase; SA, salicylic acid.

In contrast to the fungus, for which not many transcripts were observed, 599 grapevine genes were regulated due to the quiescent presence of *B. cinerea*, only 21 genes of them being down-regulated (**Figure 3C** and **Supplemental Table S3**). In this set of *Botrytis*-induced genes, functional classes related to responses to stress, amino acid metabolism for redox activity and phenylpropanoid pathways, signaling, and TFs were over-represented (**Table 2** and **Supplemental Table S10**). The visualization of individual gene responses in biotic stress pathway using MapMan tool also confirmed a remarkable induction of genes related to signaling, TFs, proteolysis, PR proteins, and secondary metabolism (**Figure 6**).

**Table 2:** Functional classes enriched in the differentially expressed genes at hard-green berry stage due to quiescent *B. cinerea,* using VitisNet.

VitisNet term	Description [# in DEG of # total]	Up-regulated genes (#)	Enrichment	*P*-value
10360	Phenylalanine metabolism [11 of 186]	11	2.90	2.08E-03
10480	Glutathione metabolism [12 of 127]	12	4.63	1.62E-05
10530	Aminosugars metabolism [7 of 76]	6	4.52	1.13E-03
10680	Methane metabolism [9 of 108]	9	4.09	4.80E-04
10940	Phenylpropanoid biosynthesis [22 of 187]	22	5.77	7.44E-11
10941	Flavonoid biosynthesis [9 of 153]	9	2.88	5.36E-03
30008	Ethylene signaling [19 of 232]	19	4.02	5.16E-07
30011	Jasmonate signaling [11 of 84]	11	6.40	1.52E-06
34626	Plant–pathogen interaction [14 of 311]	14	2.21	6.69E-03
50121	Porters cat 1 to 6 [9 of 160]	8	2.76	7.13E-03
60003	AP2 EREBP [12 of 131]	12	4.49	2.21E-05
60044	MYB [19 of 161]	19	5.79	1.39E-09
60046	NAC [9 of 75]	9	5.88	2.83E-05
60066	WRKY [17 of 62]	16	13.44	1.25E-11

About one-sixth of the differentially expressed genes were those involved in signaling, dominated by the receptor-like protein kinases (RLKs) (**Table 3** and **Supplemental Table S3**). Of the membrane-localized RLKs which were switched on during infection initiation at flowering, *Clavata1 receptor kinase* (*CLV1*), *Brassinosteroid insensitive 1-associated kinase 1* (*BAK1*), and *Wall-associated kinase 1* (*WAK1*) (Haile et al., 2017) were found induced in the hard-green berry as well during quiescent infection. However, the involvement of *WAK1*, a damage associated pattern receptor which recognizes plant cell wall–derived oligogalacturonides due to cell wall degradation (Brutus et al., 2010), during quiescent interaction was not obvious. From the transcriptional alteration of RLKs, lectin protein kinase and protein kinase 1 seem to be quiescent-stage-specific. The putative orthologues in other species are associated to plant immunity (Guidarelli et al., 2014), in particular the latter is known to mediate signaling in response to *B. cinerea* (Abuqamar et al., 2008). In addition, transcripts related to Ca^2+^ mediated signaling (such as calcium- and calmodulin-binding proteins) and oxidative stresses (mainly GST and cytochrome P450 monooxygenases) were also found to be possibly involved in the ongoing berry immunity.

**Table 3:** Selected differentially expressed grapevine genes due to *B. cinerea* infection (at 4 and 12 wpi).

ID	Fold change (log2)			Functional annotation	ID	Fold change (log2)			Functional annotation
	HG	PEG	EG			HG	PEG	EG
***Recognition and signaling***					***Cell wall***				
VIT_12s0055g01280	2.0			Brassinosteroid insensitive 1-associated receptor kinase 1	VIT_06s0004g01990	3.1			Proline-rich extensin-like family protein
VIT_12s0121g00300			1.2	Brassinosteroid insensitive 1-associated receptor kinase 1	VIT_11s0052g01220	1.9	2.5	6.1	Xyloglucan endotransglycosylase 6
VIT_03s0038g01380	1.0	1.23	1.9	Calcium-binding EF hand	VIT_06s0009g02560		8.2	15.6	Pectinesterase family
VIT_14s0030g02150	4.2			Calmodulin	VIT_09s0002g00330		-1.6	-1.6	Pectinesterase PME1
VIT_14s0006g01400		1.13	1.4	Calmodulin	VIT_08s0007g07760		5.0	8.3	Polygalacturonase PG1
VIT_11s0016g03080	2.5	1.99	1.6	Clavata1 receptor kinase (CLV1)	VIT_12s0055g00020	1.2	1.2	1.1	UDP-glucose glucosyltransferase
VIT_17s0000g07560	1.1			EDS1 (Enhanced disease susceptibility 1)	***Response to stress and secondary metabolism***				
VIT_19s0093g00110	3.1	1.23	5.1	Glutathione S-transferase 22 GSTU22	VIT_11s0052g01110	2.7	5.0	6.1	4-coumarate-CoA ligase 1
VIT_00s0253g00150	1.9	2.69	3.3	Methyl jasmonate esterase	VIT_01s0010g01960		5.8	10.8	Anionic peroxidase
VIT_04s0023g02420		1.74	2.4	Mitogen-activated protein kinase 4	VIT_03s0017g02110	3.2	1.5	4.0	Anthocyanidin 3-O-glucosyltransferase
VIT_00s0262g00090	2.6	1.18	1.3	Receptor kinase RK20-1	VIT_08s0007g06040	1.6	1.4		Beta-1,3-glucanase
VIT_17s0000g04400	3.1			Wall-associated kinase 1 (WAK1)	VIT_16s0100g00860	4.8			Chalcone synthase
VIT_17s0000g03340	1.1		1.1	Wall-associated kinase 4	VIT_03s0038g01460		1.3	3.0	Chalcone synthase
VIT_18s0001g01300			1.2	Wall-associated receptor kinase 5	VIT_05s0094g00340	2.2			Chitinase class IV
***Trascription factors***					VIT_05s0094g00360		3.4	5.1	Chitinase class IV
VIT_07s0005g03340	2.2	2.4	4.5	Myb domain protein 14	VIT_14s0066g01150	1.1	2.9	5.02	Cinnamoyl-CoA reductase
VIT_12s0028g00860	3.0			NAC domain containing protein 42	VIT_07s0031g01380	3.7			ferulate 5-hydroxylase
VIT_19s0027g00860		4.2	3.8	NAC domain-containing protein 42	VIT_14s0128g00600		5.6	5.3	Germin-like protein 3
VIT_04s0008g05760	1.8			WRKY DNA-binding protein 18	VIT_17s0000g06290	4.8			Lipase GDSL
VIT_08s0058g00690	1.6	2.2	3.7	WRKY DNA-binding protein 33	VIT_09s0002g00510		1.1	3.6	Lipase GDSL 1
VIT_08s0058g01390	1.7			WRKY DNA-binding protein 70	VIT_02s0025g04340	3.2	2.0	1.9	Osmotin
***Cell wall***					VIT_05s0077g01690	2.0	5.2	7.6	Pathogenesis protein 10
VIT_06s0004g01270			-1.7	Callose synthase catalytic subunit	VIT_03s0088g00700		3.6	2.79	Pathogenesis related protein 1
VIT_19s0014g05190			1.6	Cellulase precursor	VIT_03s0088g00710	3.5			Pathogenesis-related protein 1
VIT_00s0580g00010			-1.2	Cellulose synthase CSLC06	VIT_14s0068g01920	2.3	3.9	5.02	Peroxidase
VIT_05s0049g00010	1.2			Cellulose synthase CSLG2	VIT_16s0039g01100	4.9	2.4	3.48	Phenylalanin ammonia-lyase
VIT_14s0128g00690	4.2			Germin protein 3	VIT_00s0480g00030	2.8	2.4	2.00	Polyphenol oxidase
VIT_01s0010g02040		-1.9	-2.6	Hydroxyproline-rich glycoprotein	VIT_08s0058g00790	3.2	2.8	2.55	Secoisolariciresinol dehydrogenase
VIT_05s0020g03470			1.2	Hydroxyproline-rich glycoprotein	VIT_16s0100g01160	4.5	4.0	5.30	Stilbene synthase
VIT_11s0016g00590		-3.1	-3.7	Invertase/pectin methylesterase inhibitor	VIT_02s0025g04300	3.8	1.0		Thaumatin

The quiescent *B. cinerea* prompted the expression of key TFs playing an important role in plant–microbe interaction (**Table 3** and **Supplemental Table S3**). Most prominent was the MYB TF family: 21 genes encoding 14 different MYB proteins, including *VvMYB14* and *VvMYBPA1* which respectively regulate stilbene and proanthocyanidin biosynthesis. *VvWRKY33*, known to regulate defense response against pathogens (Birkenbihl et al., 2012; Merz et al., 2015), was also among the TFs involved in the hard-green berry response. From the NAC and WRKY TF families, it is worth mentioning the ortholog to *NAC042*, regulates phytoalexin biosynthesis (Saga et al., 2012); the ortholog to *WRKY51*, mediates the repression of JA signaling in a SA- and low-oleic-acid-dependent manner (Gao et al., 2011); and the orthologs to *NAC036*, *WRKY18* and *WRKY70*, which regulate SA biosynthesis and SA signal transduction (Wang et al., 2006) in Arabidopsis. The up-regulation of SA signaling marker genes, such as *PR1* and *EDS1*, is a further indication of SA involvement in enhancing the defense ability of the hard-green berry. In addition, the induction of 3 *ACC oxidase* and 12 *AP2/ERF* genes underlines that ET signaling was also in place during the interaction at quiescent state. AP2/ERF factors indeed play a key role in plant–pathogen interactions (Gutterson and Reuber, 2004; Licausi et al., 2013). Genes related to auxin metabolism were also modulated.

Intriguingly, differential regulation was also observed for genes encoding various PR proteins and enzymes of the phenylpropanoid pathway and of the flavonoid and stilbenoid biosynthesis during quiescent infection (**Table 3** and **Supplemental Table S3**). For some of these genes, the induction was nearly 50 fold. PR proteins and its regulator encoding genes included: *VvPR10s* and *VvWRKY33*, previously reported as involved in the defense against *B. cinerea* and *Plasmopara viticola* in grapevine (Merz et al., 2015; Haile et al., 2017); *VvPRs* encoding β -1,3-glucanase, different classes of chitinases, osmotin, thaumatin, and cystatin, known to interfere with *B. cinerea* growth (Pernas et al, 1999; Monteiro et al., 2003), .

**Figure 7:**
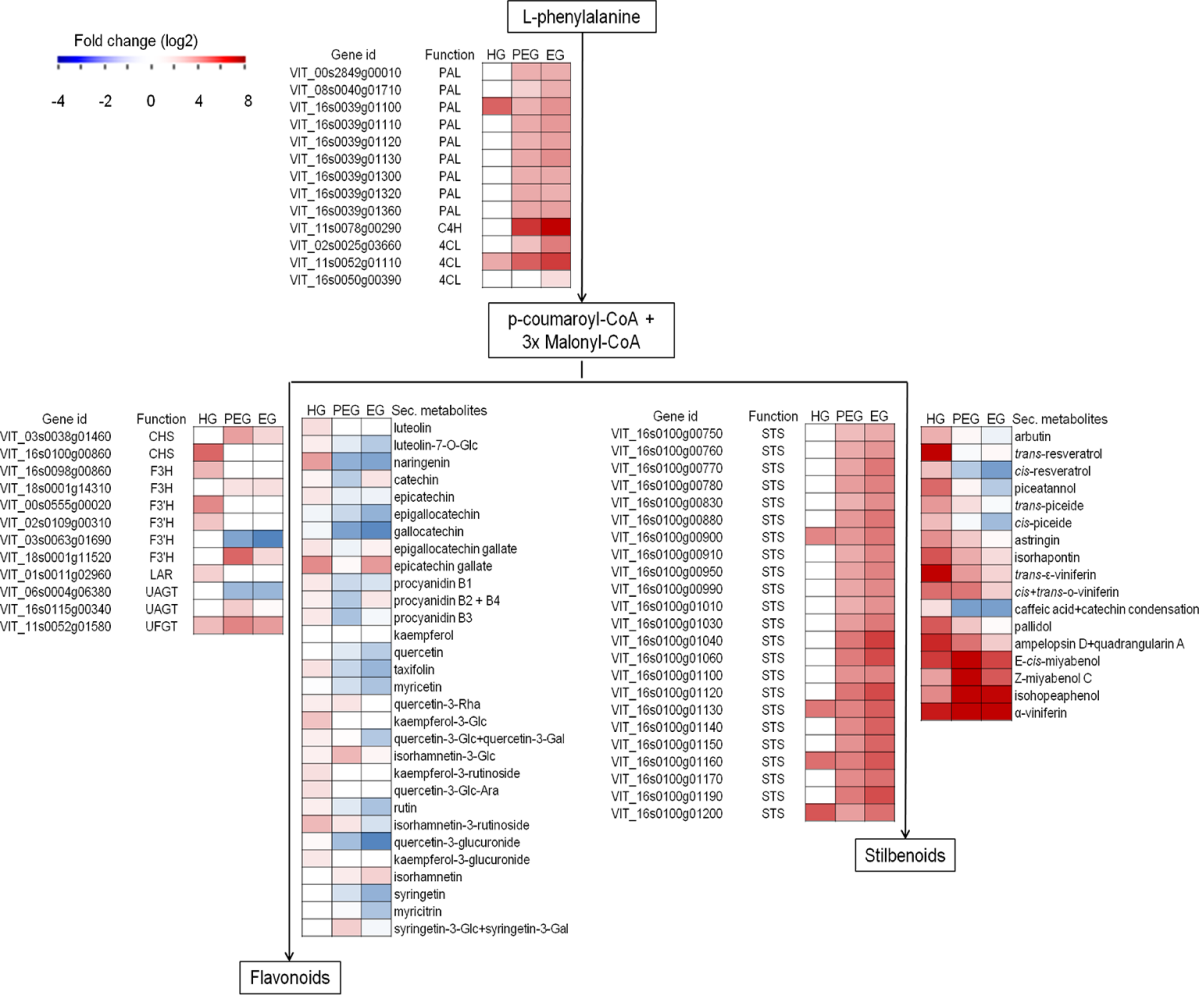
Secondary metabolism-associated genes and metabolites modulation in grapevine berries in response to *B. cinerea*. Heatmap of gene expression (from RNA-Seq result) and secondary metabolite concentration (µg/g fw, from HPLC-DAAD-MS) expressed as fold change. Fold change for secondary metabolites was computed based on the ratio of average values in *B. cinerea*- and mock-inoculated berries. HG, hard-green berry; PEG, ripe berry with pre-egressed *B. cinerea*; EG, ripe berry with egressed *B. cinerea*. Central phenylpropanoid biosynthetic pathway: PAL, phenylalanine ammonia-lyase; C4H, cinnamate 4-hydroxylase; 4CL, 4-coumarate-CoA ligase. Flavonoid biosynthetic pathway: CHS, chalcone synthase; F3H, flavonone-3-hydroxylase; F3'H, flavonoid-3'-hydroxylase; LAR, leucoanthocyanidin reductase; UAGT, UDP-glucose:anthocyanidin 5,3-O-glucosyltransferase; UFGT, UDP-glucose:flavonoid 3-O-glucosyltransferase. Stilbenoid biodynthesis pathway: STS, stilbene synthase.

Considering polyphenol biosynthesis, genes involved in monolignol, flavonoid, and stilbenoid biosynthesis pathways, such as phenylalanine ammonia-lyase, stilbene synthase, anthocyanidin 3-O-glucosyltransferase, chalcone synthase, and cinnamoyl-CoA reductase were found differentially regulated in the hard-green berry with quiescent *Botrytis* (**Figure 7** and **Supplemental Table S3**). A number of compounds of the phenylpropanoid pathway were also quantified by UHPLC-DAD-MS (**Figure 7** and **Supplemental Table S11**). The strongest effect was recorded on flavonoids and stilbenoids, in particular on compounds known to mediate defense against pathogens: resveratrol, viniferins, ampelopsin, miyabenols, isohopeaphenol, catechin, and proanthocyanidins (Jersch et al., 1989; Goetz et al., 1999; Pezet et al., 2003a; Favaron et al., 2007; Hammerbacher et al., 2011). For the stilbenoid class, most of the compounds were below the detection limit in control samples.

Taken together, even though we did not observe any known virulence related genes of the pathogen which can provoke response in the hard-green berries, it seemed, that the berries were alerted with enhanced immunity as it recognizes non-self organism, which in turn helped the berry to contain the pathogen. This gives an interesting insight that there is a molecular communication going on between the quiescent *B. cinerea* and the hard-green berry.

### Transcriptional Alterations of *B. cinerea* During Pre-Egression and Egression Stages, at Ripening

At ripening (12 wpi), two kinds of berries were collected from *Botryits*-treated samples: berries without visible *B. cinerea* outgrowth (pre-egressed *B. cinerea*) and berries with visible signs of *B. cinerea* outgrowth (egressed *B. cinerea*). Both samples were subjected to RNA-seq analysis. In the samples with pre-egressed *B. cinerea*, as in hard-green berry samples, the number of fungal transcripts was unfortunately very low, even though the sequencing depth was doubled. Moreover, in one of the biological replicates (replicate 2) the growth of the fungus was more advanced than in the rest two, as inferred from the number of fungal transcripts obtained (**Supplemental Table S2**). Taking the same threshold used in the hard-green berry samples (an average of at least ten reads) in the two biological replicates, excluding the replicate two, 431 genes were selected for functional annotation and further analysis (**Supplemental Table S7**). The most represented functional classes in this gene set are provided in **Supplemental Table S12**.

Within the 431 genes, there were several genes encoding proteins functionally associated to the infection process, among which dyp-type peroxidase and galactose oxidase, involved in generation and detoxification of ROS (Schumacher et al., 2015); polygalacturonase, deployed in pectin degradation; glyoxal oxidase and oxalate decarboxylase, both catalyzing oxalate; different types of oxidoreductases; and cerato-platanin BcSPL1, a small protein required for full virulence (Frías et al., 2011; Frías et al., 2014). Interestingly, these genes showed a low number of raw reads or were not expressed at all at 4 wpi (**Table 4**), indicating that the fungus was in a different physiological state in the ripe berries before egression as compared to that in the hard-green berries, though there was no any apparent disease symptom in both cases.

**Table 4:** Selected *B. cinerea* genes having more RNA-seq reads at pre-egression (12 wpi, on ripe berry) than at quiescence (4 wpi, on hard-green berry).

Gene ID	Function (Blast2GO)	Further description	RNA-seq reads (average)	
			4 wpi	12 wpi (Peg)*
Bcin13g05720	Dyp-type peroxidase	BcPRD1, Dyp-type peroxidase	2	42
Bcin13g05710	Galactose oxidase beta-propeller	BcGOX1, Galactose oxidase	4	30
Bcin12g02040	Acid protease	BcAP8, aspartic proteinase	1	12
Bcin14g00850	Polygalacturonase	Pectin degradation	0	30
Bcin06g01930	Glyoxal oxidase	Glyoxal oxidase	3	31
Bcin15g02380	Acid protease partial	Glutamic protease	0	42
Bcin03g00500	Probable rot1 PRECURSOR	Cerato-platanin family protein BcSpl1	5	29
Bcin01g11220	Glycoside hydrolase family 17	b-1,3-Glucosidase	4	16
Bcin07g00160	Glycoside hydrolase family 18	CAZyme	5	42
Bcin04g05650	Oxalate decarboxylase family Bicupin		1	41
Bcin11g02720	Aldo keto reductase	Oxidoreductase	9	35
Bcin11g02630	Phytanoyl- dioxygenase	Oxidoreductase	0	23
Bcin15g03620	Glycosyltransferase family 35	CAZyme	8	20
Bcin11g06080	ATP synthase H mitochondrial precursor		3	40

*The sequence depth of the samples was double than 4 wpi.

Unlike, in the egressed samples, 86% of total *B. cinerea* transcriptome was expressed, about 10,000 of the total 11,701 predicted genes (van Kan et al., 2016). Such a massive transcriptional activity was not seen in the other infection stages, possibly due to the low amount of the fungus, but likely also to a reduced transcriptional activity at those stages. In other words, at ripening, the time, the status of the host tissue, and the environmental conditions, components of the disease pyramid, were conducive for *B. cinerea* to egress and grow vigorously, as observed in **Figure 1C**.

Compared to the transcriptional changes of *B. cinerea* cultured in PDB, 3,548 genes were differentially regulated during egression at ripening (**Figure 3C** and **Supplemental Table S5**). These DE genes are over-represented in metabolic processes, ion binding, catalytic and oxidoreductase activities, cytoplasm, intracellular part functional classes (**Supplemental Table S13**). Genes encoding carbohydrate-active enzymes and others involved in plant cell wall degradation (Espino et al., 2010; Blanco-Ulate et al., 2014), such as *Bcin10g06130* and *Bcin14g01630*, encoding pectinases, *Bcin03g01680*, encoding a polygalacturonase, and *Bcin07g06480* and *Bcin15g03080*, encoding cutinases, were expressed more during egression than in PDB medium. Other virulence and/or growth related genes having similar expression trend as those mentioned above were: ROS producers and scavengers like *Bcin03g03390*, *Bcin13g05710*, and *Bcin13g05720* (Rolke et al., 2004; Schumacher et al., 2015); characterized aspartic proteases *Bcin12g02040* and *Bcin12g00180* (ten Have et al., 2010); membrane transporters, mostly the ATP-binding cassette; and the botcinic acid and botrydial phytotoxins *Bcin12g06390* and *Bcin12g06380* (Siewers et al., 2005; Dalmais et al., 2011). On the other hand, known virulence genes like *BcPG1* (*Bcin14g00850*), *BcGST1* (*Bcin10g00740*), *BcBOA6* (*Bcin01g00060*), and *BcSPL1* (*Bcin03g00500*) had similar or lower expression level during egression as compared to PDB cultured *Botrytis*. This, however, does not mean that they have not played any role in the necrotrophic stage of infection during egression at ripening, as the number of reads of these genes was reasonably high at this stage (**Table 5**).

**Table 5:** RNA-seq reads of key *B. cinerea* virulence genes which were not considered as differentially expressed in comparison to PDB culture of *B. cinerea*.

Gene ID	Gene name	RNA-seq reads (average)	
		Eg	PDB culture
Bcin14g00850	Polygalacturonase1 (BcPG1)	24,289	147,821
Bcin10g00740	Glutathione S-transferase (BcGST1)	1,496	2,655
Bcin01g00060	Botcinic acid6 (BcBOA6)	740	1,048
Bcin03g00500	Cerato-platanin family protein (BcSPL1)	29,201	64,662

### Response of Ripe Berries to *B. cinerea*, at Pre-Egression and Egression States

Grapevine berries responded to the necrotrophic colonization of the fungus, during egression, by reprogramming the transcription of 2,213 genes (**Figure 3C** and **Supplemental Table S4**). Of these genes, 1,564 were already differentially regulated in pre-egressed samples. The GO enrichment analysis performed on the DE genes of pre-egressed and egressed samples showed a high overlap in the enriched biological processes: secondary metabolic process, response to stimulus, catabolic process, and transport were among the shared enriched functional classes by both samples (**Supplemental Table S14**).

Besides the very high overlap of DE genes of the pre-egressed and egressed samples, there existed also a general similarity in the expression trend (up- or down-regulation) of these common genes shared by both samples (**Figure 3C** and **Supplemental Table S4**). As a result, we used the DE genes of the egressed stage to visualize the biotic stress pathway (**Figure 6**), *via* MapMan, as it can give enough insight of the pre-egressed stage as well. **Figure 6** showed that the transcriptional changes involved in biotic stress were huge. Although it might seem surprising, it is not uncommon to see such an extensive modulation of defense related genes in infected plant tissues also when the pathogen invades the plant host tissues (for example in: Alkan et al., 2014; Agudelo-Romero et al., 2015; Kelloniemi et al., 2015). This could be due to the futile attempts of the infected tissue reacting against the pathogen and/or to the transcriptome attributes of other cell layers, not yet colonized, as it is very difficult to spatially resolute the tissues where the pathogen is growing from those which are not yet colonized. The comparison of the two samples highlights the presence of several genes associated to auxin, JA and ET signaling and cell wall modification uniquely modulated in the ripe berry in response to *B. cinerea* (**Figure 6**).

Hormones interactions during plant defense are extremely complex. At transcriptional level, it appeared that auxin, ET, JA, and SA were involved (**Supplemental Table S4**). However, the co-activation of SA and JA pathways is uncommon and very interesting (Oirdi et al., 2011; Kelloniemi et al., 2015) and therefore should be further investigated. Genes encoding PR1, a SA marker, and ZIM-domain1, a JA marker, were all induced (**Table 3** and **Supplemental Table S4**).

Polyphenol biosynthesis pathway was also affected by the egression of *B. cinerea* at ripening (**Table 3** and **Supplemental Table S4**), though the transcription of genes encoding dihydroflavonol-4-reductase and flavanone 3-hydroxylase, both involved in flavonoid biosynthesis, was not affected. In line with this evidence, almost all of the quantified flavonoids were significantly lower or not significantly different in the berries with egressed *B. cinerea* as compared to the control (**Figure 7** and **Supplemental Table S15**). On the other hand, genes encoding other key enzymes such as cinnamate 4-hydroxylase, 4-coumarate-CoA ligase 1, and stilbene synthase which are involved in the biosynthesis pathway were induced. With regard to stilbenoid content, the concentration of piceide, miyabenol, viniferins, and isohopeaphenol was high in berries with pre-egressed *Botrytis*, intermediate in berries with egressed *Botrytis*, and very low in healthy berries (**Figure 7** and **Supplemental Table S15**).

Most of the genes involved in monolignol biosynthesis (such as cinnamoyl-CoA reductase and cinnamyl alcohol dehydrogenase) were differentially regulated (**Supplemental Table S4**), unlike a few but important genes like caffeoyl-CoA O-methyltransferase (CCoAMT) and ferulate 5-hydroxylase (F5H) encoding ones, which were not differentially regulated. CCoAMT is involved in ferulic esterification and lignification process in response to pathogen attack in grapevine (Busam et al., 1997). From the polyphenol analysis, caffeate and ferulate, substrate for CCoAMT were not detected in any of the samples at ripening (**Supplemental Table S15**). This perhaps might suggest that lignification is not part of plant defense component during ripening. Rather, a number of genes encoding proteins involved in ripening-associated cell wall extensibility and disassembly like xyloglucan endotransglucosylase and polygalacturonase and pectinesterases (Nunan et al., 2001; Deytieux-Belleau et al., 2008) were highly upregulated in ripe berries with pre-egressed and egressed *Botrytis* (**Supplemental Table S4**). Pectin methylesterases (PMEs) are suggested to be involved both in cell wall loosening and strengthening (Micheli, 2001), in particular PMEs are involved in the de-esterification *in muro* of pectin which becomes more susceptible to the degradation by pectic enzymes secreted by *B. cinerea* during the initial stages of infection (Lionetti et al., 2012). However it seemed here that cell wall loosening prevailed over cell strengthening as there were a lot of induced polygalacturonases which degrade polygalacturonans, made accessible by the pectinesterases.

## Discussion

**Figure 8:**
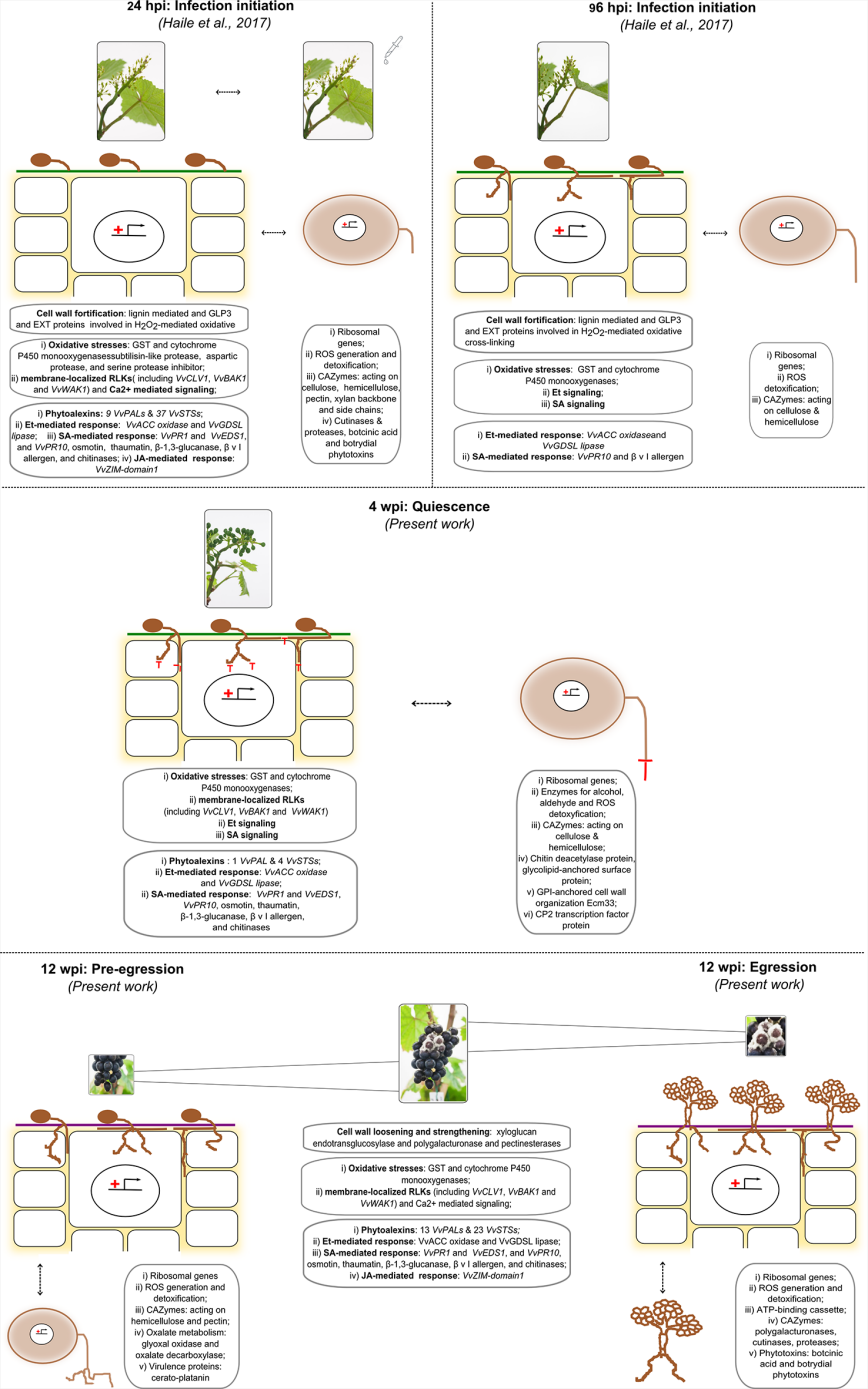
Depiction of the most important events from infection initiation (24 hpi) till fungal egression (12 wpi) passing through entering quiescence (96 hpi) and quiescence (4 wpi) phases occurring both in the grapevine and in the fungal cells, respectively. The events at the initial stages (24 and 96 hpi) are taken from Haile et al. (2017), while the other came out from the present work.

In a previous study, grapevine flowers were challenged by placing suspension of *B. cinerea* conidia to induce infection (Haile et al., 2017). The results showed that fungal genes encoding virulence factors and proteins known to contribute to the infection program were highly induced. Consequently, the flower reprogrammed its transcriptome which resulted in increased expression levels of genes involved in reduction–oxidation processes, genes encoding antimicrobial proteins, and genes of the polyphenol biosynthesis pathway, for the production of phytoalexins and precursors for cell wall toughening (Haile et al., 2017). These defense reactions of the flower appeared to be able to put *B. cinerea* into quiescence. To know more of the later stages of the infection process, this study was conducted with an in-depth look at the molecular communication between the fungus and the berry at hard-green (4 wpi) and at ripe (12 wpi) stages. **Figure 8** summarizes the most important events occurring from infection initiation (24 hpi) till fungal egression (12 wpi) passing through entering in quiescence (96 hpi) and quiescence (4 wpi) phases occurring both in the grapevine and in the fungal cells, respectively.

In the very early stage of quiescence in the flowers, ribosomal genes were prevalent in the *in planta* expressed *B. cinerea* genes (Haile et al., 2017). A similar high proportion of ribosomal genes were also observed within the *in planta* expressed genes of *B. cinerea* in the hard-green berry at 4 wpi (**Supplemental Table S6**). Nevertheless, there was no known virulence-related gene in the *Botrytis* genes expressed *in planta* at 4 wpi. Yet, other biological activities helping the fungus to stay “alive” were likely on-going since elongation factors, ATP synthesis, and ATP-dependent molecular functions related genes were transcriptionally active. For example, *Bcin15g02120* (glyceraldehyde-3-phosphate dehydrogenase) and *Bcin16g04800* (malate dehydrogenase) genes involved in glycolysis and tricarboxylic acid cycle for energy metabolism were expressed. Also among the expressed genes were an ATP-dependent cell division cycle protein 48 (p97/valosin-containing protein, *Bcin08g03700*) gene, involved in cell cycle and transcriptional regulation (Wang et al., 2004), and a number of ATP-dependent membrane transporter genes. As to the source of energy, the quiescent *B. cinerea* expressed 34 CAZyme genes suggesting that also in this phase it is still capable of extracting energy from the host.

The absence of detectable pathogenic progression of the quiescent *B. cinerea* is possibly due to the continued induction at 4 wpi of those transcripts that were found involved in plant defense at bloom at initial infection stage (Haile et al., 2017). Such continued activation of defense pathways between appressoria formation and quiescent stages of infection was also reported in unripe green tomato infection by *Colletotrichum gloeosporioides* (Alkan et al., 2014). The expressions of *VvWRKY33* gene, also correlates with the expression of *VvPR10* genes in response to defense in grapevine (Merz et al., 2015; Haile et al., 2017), and genes of different families of PR proteins, including PR10, were highly induced in the hard-green berry due to the quiescently present *B. cinerea* (**Supplemental Table S3**). The grapevine *WRKY33* functional homologue, *AtWRKY33,* has been shown to be involved in response to biotic and abiotic stresses (Zheng et al., 2006; Jiang and Deyholos, 2009; Li et al., 2011; Birkenbihl et al., 2012). On the other hand, five GDSL lipase encoding genes, whose expression was not affected during flower infection, were strongly upregulated (up to 25-fold) during quiescent infection. No previous report associated these lipases with a defense response against pathogens; however, other lipases, such as GDSL Lipase 1 (Oh et al., 2005), are involved in defense against *Alternaria brassicicola* and *B. cinerea* in *Arabidopsis* in an ET dependent manner regulated by *WRKY33* (Oh et al., 2005; Kwon et al., 2009; Birkenbihl et al., 2012).

The induction of PR proteins in the hard-green berry might compel cell wall remodeling in the quiescent *B. cinerea*. The qPCR assay confirmed that *Bcin11g04800*, a gene encoding chitin deacetylase, was highly induced during the quiescent phase (**Figure 5**). Chitin deacetylation is a mechanism used by plant pathogens as well as endophytic fungi to protect their cell wall from being attacked by plant chitinases (Deising and Siegrist, 1995; El Gueddari et al., 2002). Chitin depolymerization into deacetylated chitosan oligomers avoid the binding by plant receptors and the consequential plant immune responses (Petutschnig et al., 2010; Liu et al., 2012). Recently, Cord-Landwehr and colleagues (Cord-Landwehr et al., 2016) demonstrated that chitosan oligomers, deacetylated chitin extracted from an endophytic fungus *Pestalotiopsis* sp., were not able to elicit plant immunity in rice cell suspension culture. Thus, the enzyme might play an important role, particularly during quiescent phase, to impair the recognition of the quiescent *B. cinerea* by the plant immunity system. In addition to chitin deacetylase, other genes encoding glycolipid-anchored surface protein and GPI-anchored cell wall organization ECM33, which in yeast are linked to cell wall integrity to ensure viability (Pardo et al., 2004), were also expressed during the quiescent phase, suggesting that the fungus is also actively defending itself besides the basal metabolic activity.

The activation of stilbenoid and flavonoid biosynthetic pathways by grapevine in response to active pathogenic infection is well documented (Langcake, 1981; Jeandet et al., 1995; Keller et al., 2003; Malacarne et al., 2011; Agudelo-Romero et al., 2015; Kelloniemi et al., 2015; Haile et al., 2017). Here we observed that genes encoding essential enzymes of the pathways, such as stilbene synthase, chalcone synthase, flavanone 3-hydroxylase, and anthocyanidin 3-O-glucosyltransferase, were actively engaged during quiescent infection. A key transcription factor regulating stilbene biosynthesis *VvMYB14* (Höll et al., 2013) was also modulated. As expected, from the transcriptional analysis results, several polyphenols were also at higher concentration in the inoculated samples. The content of resveratrol and its monomeric (for example astringin, isorhapontin, and piceide) and oligomeric (for example miyabenol, isohopeaphenol, and viniferin) derivatives, known defense compounds Pezet et al., 2003a; Favaron et al., 2007; Hammerbacher et al., 2011), was higher in hard-green berry with quiescent *Botrytis* than in the control samples.

Relevant transcripts for the synthesis of monolignol precursors (*VvPAL*, *VvCOMT*, *VvCCoAMT*), which increase penetration resistance in the plant cells (Bhuiyan et al., 2009), and other lignin forming enzymes like GLP3 and EXT, were also induced in the hard-green berry. Lignification at the penetration site is one of the major defense mechanisms that plants adopt to stop *B. cinerea* progress (Cantu et al., 2009; Kelloniemi et al., 2015; Haile et al., 2017). It is interesting to observe the pathway being active at 4 wpi in the hard-green berry.

However, egression of *B. cinerea* was observed after bunches were bagged for 2 weeks, starting at full coloring (approximately 10 wpi), to create high humidity around the bunch (**Figure 1C**). At the very start of the egression process, an outgrowth of *B. cinerea* (or egression) was observed on about 40% of the berries.

During egression about 86% of the *B. cinerea* transcriptome was expressed, and it encompassed genes functionally annotated as ROS producers and scavengers, CWDE, proteases, and enzymes involved in the synthesis of phytotoxic secondary metabolites, which are sustaining *B. cinerea* pathogenicity (as reviewed in Nakajima and Akutsu, 2014). *Botrytis* transcripts belonging to these functional classes were also shown to be involved in the successful infection of ripe grapevine berries and other hosts (De Cremer et al., 2013; Smith et al., 2014; Kelloniemi et al., 2015). However, the expression level of *BcPG6* and *BcPEL-like1*, pectinases which were extremely induced during initial infection at bloom (Haile et al., 2017), was much less both at ripe and in PDB culture. In general, as expected, the transcriptional activity of the pathogen was high during egression. An important question is what signals and/or environmental changes made the quiescent *B. cinerea* egressing. Although still speculative, it is likely that ripening associated signals and physical and chemical changes play an important role in triggering the transition from the prolonged quiescent to the egression phase.

We have noticed that a lot of grapevine genes involved in cell wall disassembly were induced. Cell wall loosening, cuticular changes, conversion of acids into sugars, and a steadily diminishing of antifungal compounds are reported to favor pathogen egression (Prusky, 1996; Prusky et al., 2013). It has also been shown that the protective role of berry cuticle on *B. cinerea* infection decreased with ripening (Commenil et al., 1997; Mlikota-Gabler et al., 2003). The structural changes in the cell wall polysaccharides that lead to fruit softening could cause susceptibility to necrotrophic pathogens at fruit ripening (Cantu et al., 2008). These authors showed that suppression of the ripening-associated cell wall loosening genes reduced the susceptibility of ripe tomato to *B. cinerea* (Cantu et al., 2008). Besides cell wall loosening, sugars and organic acids exudates appearing on ripe berry surface have also the potential to stimulate and promote *B. cinerea* outgrowth (Padgett and Morrison, 1990; Pezet et al., 2003b; Kretschmer et al., 2007). Furthermore, a shift in plant-hormone synthesis and signaling balance happening during ripening also trigger fungal pathogenicity factors (Prusky, 1996; Prusky et al., 2013).

Considering the transcriptional alterations underwent in the ripe berry, as a response to *Botrytis* egression, a wide array of defense responses were noticed, suggesting that the tissue under colonization “never gives up” rearranging its defense mechanisms. With regard to stilbenic compounds, surprisingly oligomerization was apparently driven by the presence of the fungus. Oligomerization, according to Pezet et al. (2003a) and Malacarne et al. (2011), increases toxicity. The amount of oligomeric stilbenoids was very little in the control berries and higher in the treated berries especially in the pre-egressed ones. One possible explanation is the fact that the *B. cinerea* LACCASE 2 enzyme (encoded by *Bcin14g02510*) which oxidizes resveratrol (Schouten et al., 2002) was extremely induced (128 fold) in the egressed *Botrytis*.

Last, the evolution of the berry skin tissue is an important component of the berry-*Botrytis* interaction. It has been noticed that the extent of the expression of cell wall modifying genes increases toward maturity. It is actually a phenological cue that once the seeds are matured, cell wall loosening occurs. The differential accumulation of xyloglucan endotransglucosylases, involved in cell wall extensibility (Miedes et al., 2011) and polygalacturonases and pectinases, involved in berry softening (Deytieux-Belleau et al., 2008), are high during berry ripening (Nunan et al., 2001; Lijavetzky et al., 2012). These cell wall modifying genes were remarkably induced in ripe berries with both pre-egressed and egressed *B. cinerea*, suggesting that the fungus took advantage of the onset of the fruit cell wall self-disassembly, exploiting endogenous developmental programs to activate its own virulence CWDE. In tomato, the expression of the ripening associated genes polygalacturonase and expansin have been shown to facilitate susceptibility to *B. cinerea* (Cantu et al., 2008; Cantu et al., 2009). It has also been suggested that the fungus can induce unripe fruit cell wall-modifying proteins in order to increase fruit susceptibility (Cantu et al., 2009). We, however, haven’t observed any hastening of ripening process in *Botrytis*-inoculated samples. Both mock- and *Botrytis*-inoculated bunches ripen on similar time after inoculation.

Unlike in the flower response to *Botrytis* infection, in ripe berries with egressed *Botrytis* we did not observe a regulation of monolignol related genes such as CCoAMT and F5H, involved in cell wall apposition in response to pathogen attack (Busam et al., 1997; Bhuiyan et al., 2009), strongly suggesting there was no cell wall fortification.

In conclusion, *B. cinerea* inoculated at bloom was quiescent for 12 weeks and egressed at ripening, suggesting that the defense responses of the berries were efficient to halt the fungal growth only until maturity. Our study revealed that the defense responses of immature berries (at 4 wpi) that kept *B. cinerea* quiescent were similar to the response of the plant to the pathogen at bloom (Haile et al., 2017). During this period, the fungus had cryptic interaction with the berry keeping its basal metabolic activities and deacetylating its cell wall. However, at ripe (at 12 wpi) the pathogen managed to egress and cause bunch rot, using the advantage of the fruit cell wall self-disassembly and fulfillment of other conditions (including humidity). Consequently, there were several defense responses except cell wall strengthening by the ripe berries, but not effective in halting the pathogen from berry colonization.

## Data Availability Statement

The datasets generated for this study can be found in the NCBI Short Read Archive, BioProject accession code PRJNA414966.

## Author Contributions

ZH conceptualised the project, carried out the lab work and most of the analyses, and drafted the manuscript. GM supported in the interpretation of the data and drafted the manuscript. SP supported in the interpretation of the data. PS, MM, and KE supported in the transcriptomic data analysis. DM and UV carried out the metabolic analysis and supported in the interpretation of the results. EB conceptualised the project and contributed to the discussion of the results. CM conceptualised as well as coordinated the project, contributed to the discussion of the results and to the drafting of the manuscript. All authors read and approved the final manuscript.

## Conflict of Interest

The authors declare that the research was conducted in the absence of any commercial or financial relationships that could be construed as a potential conflict of interest.
