# Supplementary figures and images for: Dual Transcriptome and Metabolic Analysis of Vitis vinifera cv. Pinot Noir Berry and Botrytis cinerea During Quiescence and Egressed Infection
Source: Front Plant Sci. 2020 Jan 30;10:1704. doi: 10.3389/fpls.2019.01704 (PMC7002552; doi:10.3389/fpls.2019.01704)

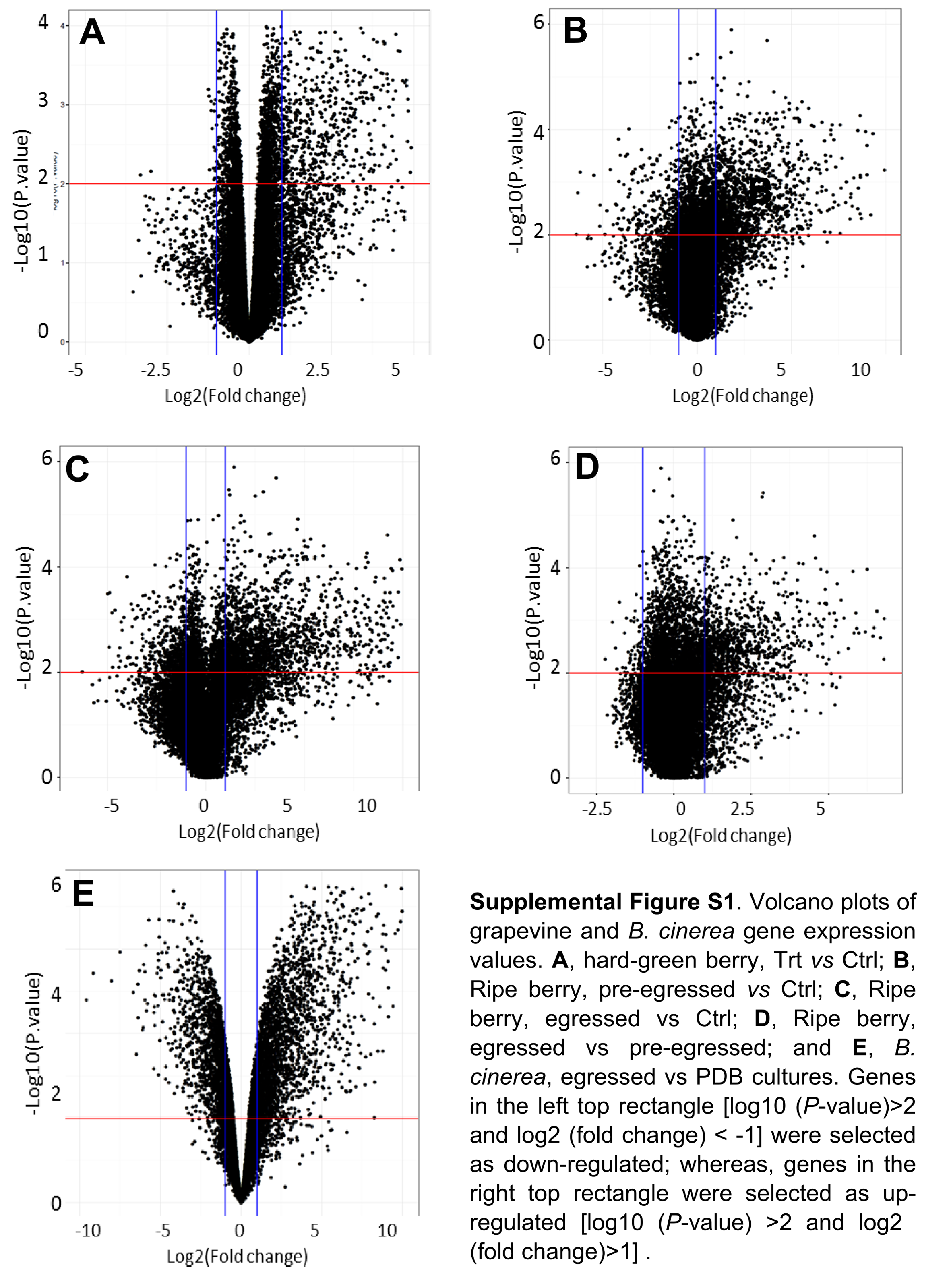

Supplement: Supplementary file 2 [file Image_1.tif]
